# Eventration of diaphragm with gastric volvulus: a case report

**DOI:** 10.1186/1757-1626-1-404

**Published:** 2008-12-17

**Authors:** Naveed Nazir Shah, Mir Mohsin, Syed Quibtiya Khursheed, Syed Suraiya Arjumand Farooq, Arshad Altaf Buchh, Abdul Quadir Quraishi

**Affiliations:** 1 Department of Surgery, Jawaharlal Nehru Medical College Hospital, AMU, Aligarh, UP, India; 2 Department of Pulmonology, Jawaharlal Nehru Medical College Hospital, AMU, Aligarh, UP, India; 3 Department of Surgery SMHS Hospital, Srinagar, Kashmir, J&K, India

## Abstract

**Background:**

Eventration of diaphragm associated with gastric volvulus is an uncommon condition.

**Case presentation:**

We are reporting a case of a 60-year-old male having left sided total diaphragmatic eventration associated with chronic intermittent organo-axial gastric volvulus. The patient presented with progressive dyspnea and intermittent gastrointestinal symptoms. Plication of left hemidiaphragm with anterior gastropexy was performed through an abdominal approach. Postoperatively the patient's symptoms improved.

**Conclusion:**

Symptomatic gastric volvulus associated with diaphragmatic eventration is a surgical emergency and always requires surgical repair.

## Background

Eventration of diaphragm is defined as an abnormal elevation of an intact diaphragm and most often is characterized by a developmental abnormality of the diaphragm musculature [1]. It usually remains asymptomatic in early life and presents later with respiratory and occasionally gastrointestinal complications. The abnormally wide subdiaphragmatic space provides the potential for abnormal rotation of stomach around itself. This abnormal rotation is known as gastric volvulus. It is described as organoaxial, if rotation occurs along the longitudinal axis connecting the gastro-esophageal junction with the pylorus so that the antrum moves from an inferior to superior position; mesentero-axial volvulus is defined as rotation along vertical axis extending from the liver to greater curvature.

We present a case of 60 year old male with eventration of left hemidiaphragm with chronic intermittent organo-axial gastric volvulus.

## Case presentation

**Figure 1:**
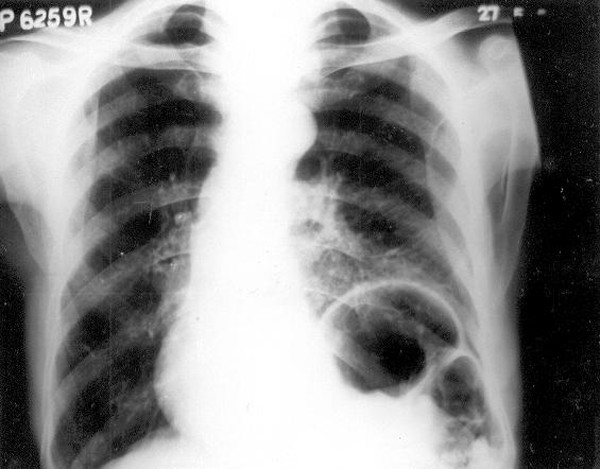
Chest roentgenogram revealing an elevated left hemidiaphragm and a shift of the lower mediastinum to right.

A 60 years old asian male, labourer presented with progressively increasing shortness of breath for last 3 years that was more in the supine position. The patient had history of cough with expectoration off and on, for last 5 years. Patient had a history of smoking (40 pack years). There was a past history of episodes of upper abdominal discomfort, early satiety off and on for which he took symptomatic treatment. There was no history of chest pain or atopy. On examination, vitals were stable. Chest examination revealed a hyperinflated chest. Diaphragmatic excursion on left side was decreased on percussion. Auscultation revealed vesicular breath sounds with prolonged expiration and decreased breath sounds on left side. Pulmonary function test (PFT) revealed a mixed obstructive – restrictive pattern. Total Lung Capacity (TLC) was 85% of predicted, Vital Capacity (VC) was 75% predicted and FEV_1 _was 60% predicted. Forced Residual Capacity (FRC) and the ratio of Forced Expiratory Volume in 1 sec (FEV_1_) and Forced Vital Capacity (FVC) i.e. FEV_1_/FVC were normal. Chest roentgenogram revealed an elevated left hemidiaphragm and a shift of the lower mediastinum to right (Figure 1). The patient improved on bronchodilators. On fluoroscopy, Sniff Test revealed a paradoxical movement of > 2 cm of left hemidiaphragm.

**Figure 2:**
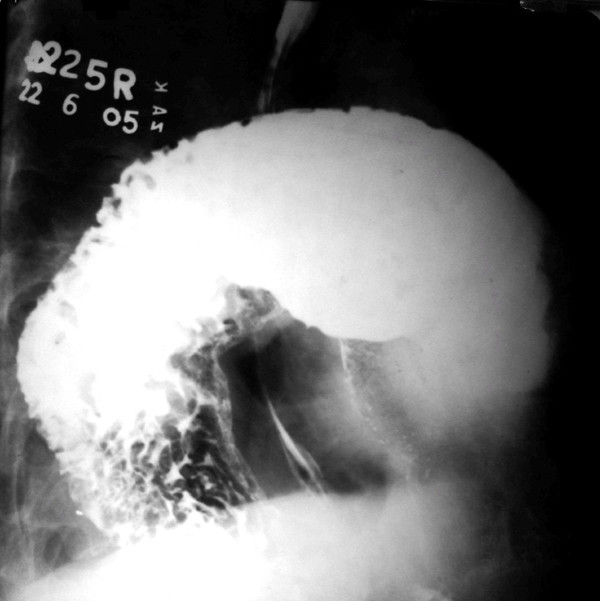
Barium study showing organoaxial type of gastric volvulus.

Three days later patient developed an episode of upper abdominal discomfort with recurrent retching and production of little vomitus. Barium study revealed organoaxial type of gastric volvulus (Figure 2).

Nasogastric tube decompression was done and patient's symptoms improved. Computed Tomography (CT) upper abdomen after 24 hours revealed total eventration of left hemidiaphragm and stomach in its normal position. A diagnosis of total eventration of left hemidiaphragm with chronic intermittent gastric volvulus was made.

Plication of left hemidiaphragm with anterior gastropexy was done via an abdominal (left subcostal) incision. Dyspnoea improved, PFT postoperatively showed slight improvement in VC, FVC, FEV_1_, FEV_1_/FVC and marked improvement in total lung capacity (TLC).

## Discussion

Eventration is a congenital anomaly of the diaphragm characterized by muscular aplasia and subsequent abnormal elevation of an intact hemidiaphragm. Pathologically, a totally eventrated hemidiaphragm consists of a thin membranous sheath attached peripherally to normal muscle at points of origin from the rib cage. It occurs almost exclusively on the left side, a point that may be of value in its differentiation from diaphragmatic paralysis.

Gastric volvulus was first described by Berti in 1866 in a female autopsied patient, and the first operation was performed by Berg in 1897.[2] Since then, over 400 cases have been reported in literature; at least 2/3^rd ^are of chronic or recurring type.[3] The reported frequency of a gastric volvulus during barium X-ray studies was 0.15%.[4] The stomach is relatively fixed at the esophageal hiatus and pylorus and is prevented from abnormal rotation by 4 gastric ligaments. Absence or weakness of these anatomic anchors results in the mobility of the stomach within the wide subdiaphragmatic space and potential for gastric volvulus.[5]

Most cases of gastric volvulus occur in association with eventration of left hemidiaphragm or a hiatus hernia. Organoaxial rotation is the most common type accounting for greater than 60% of the cases, followed by mesenteroaxial. A combined organo-axial and mesenteroaxial rotation is the least common type. Primary gastric volvulus, making up one third of cases, occurs when the stabilizing ligaments are too lax as a result of congenital or acquired causes. Secondary gastric volvulus, making up the remainder of cases, occurs in association with a paraesophageal hernia or other congenital or acquired diaphragmatic defects.[6] Diaphragmatic eventration is an uncommon consequence of blunt trauma and is often overlooked unless there is a high index of clinical suspicion.[7] Patients with unilateral diaphragmatic eventration are asymptomatic, however, some complain of dyspnea on effort or rarely orthopnea, due to the decrease in ventilation and oxygenation because of paradoxical motion of the affected diaphragm during inspiration and expiration. The severity of either symptom depends on presence or absence of underlying pulmonary disease.

Gastrointestinal (GI) symptoms may even predominate when related to volvulus of the stomach with intermittent or complete outlet obstruction.[1] Symptoms of diaphragmatic eventration may include nausea, heartburn, early satiety, post postprandial vomiting, constipation and epigastric discomfort. The classic triad of retching, severe and constant epigastric pain, and difficulty in passing a nasogastric tube should suggest the presence of acute gastric volvulus.[8]

Few case reports have shown that delayed onset of secondary gastric volvulus associated with congenital diaphragmatic eventration is also possible. [9]

The diagnosis of diaphragmatic eventration associated with gastric volvulus is usually straight forward and can be established by history and, in most cases, by routine chest X-rays and either upper GI series or CT scan.

Management of diaphragmatic eventration varies greatly depending on whether the diagnosis is made in infants or adults. Simple cases of diaphragmatic eventration may not require surgical intervention if it is not intruding significantly into the thoracic cavity and is not associated with adverse symptoms.[1] However treatment by plication is indicated if there are symptoms of dyspnea, recurrent pneumonia, chronic bronchitis, chest pain, poor exercise tolerance and functional disorders of stomach. [10] Symptomatic gastric volvulus associated with diaphragmatic eventration is a surgical emergency and always requires surgical repair. [5] The most widely accepted approach is repair via an abdominal (subcostal) incision because this allows ready access to both diaphragms for plication, permits anterior gastric fixation via a gastropexy or gastrostomy, and allows abdominal exploration for associated gastrointestinal anomalies.[5] Recently, endoscopic reduction of the gastric volvulus has been reported [11], and reports of percutaneous endoscopic gastropexy (PEG) [12] and laparoscopic gastropexy [13] for a gastric volvulus have increased.

## Consent

Written informed consent was obtained from the patient for publication of this case report and accompanying images. A copy of the written consent is available for review by the Editor-in-Chief of this journal.

## Competing interests

The authors declare that they have no competing interests.

## Authors' contributions

NNS and MM made substantial contributions to conception and design, acquisition of data and drafting the manuscript. SQK, SSAF, AAB and AQQ contributed significantly in acquisition of data, in drafting the manuscript and revising it.
